# Green space visits among Turkish and South Asian Surinamese women with a high cardiometabolic risk living in disadvantaged neighborhoods in the Netherlands: motives, means and prerequisites

**DOI:** 10.1186/s12939-024-02344-8

**Published:** 2024-12-02

**Authors:** Lieke van den Brekel, Helene R. Voogdt-Pruis, Lian Wispelweij, Laxmie Jawalapershad, Soerinder Narain, Kerstin Klipstein-Grobusch, Diederick E. Grobbee, Virissa Lenters, Joreintje D. Mackenbach, Ilonca Vaartjes

**Affiliations:** 1 Department of Epidemiology and Health Economics, Julius Center for Health Sciences and Primary Care, Utrecht University Medical Center, Utrecht University, Utrecht, The Netherlands; 2 Department of Global Public Health and Bioethics, Julius Center for Health Sciences and Primary Care, University Medical Center Utrecht, Utrecht University, Utrecht, The Netherlands; 3 Stichting Ester, The Hague, The Netherlands; 4 Stichting Vobis, The Hague, The Netherlands; 5 Division of Epidemiology and Biostatistics, School of Public Health, Faculty of Health Sciences, University of the Witwatersrand, Johannesburg, South Africa; 6 Amsterdam Institute for Life and Environment, Vrije Universiteit Amsterdam, Amsterdam, The Netherlands; 7 Department of Epidemiology and Data Science, Amsterdam UMC, Vrije Universiteit Amsterdam, Amsterdam, The Netherlands; 8 Upstream Team, Amsterdam, The Netherlands; 9 Amsterdam Public Health Research Institute, Amsterdam, The Netherlands

**Keywords:** Green space, Ethnicity, Turkish, Surinamese, Health disparities, Equity, Environment

## Abstract

**Background:**

The use of urban green spaces differs by social characteristics, including gender, ethnicity, and socioeconomic position. We examined motives, means and prerequisites to visit green space of marginalised populations with high cardiometabolic risk in the Netherlands, namely women with a Turkish or South Asian Surinamese background residing in disadvantaged neighbourhoods.

**Methods:**

We conducted six focus group discussions in two Dutch cities. The study was performed in collaboration with social workers from the local communities with similar ethnic backgrounds as the participants. A thematic analysis was carried out.

**Results:**

Sixteen Turkish women and 30 South Asian Surinamese women participated. Motives, means and prerequisites that emerged covered four themes: social, personal, environmental characteristics and undertaking activities. Socializing was an important motive to visit green space. Personal motives mainly consisted of positive effects on mental and physical well-being. Activities undertaken in green space were often a means to socialize or improve well-being. Many environmental factors, including safety, aesthetics, and (sanitary) facilities, influenced motivation to visit green space. Except for environmental characteristics, motives, means and prerequisites largely overlapped between ethnic groups. There were notable interactions between the themes.

**Conclusion:**

Motives, means and prerequisites to visit green space of women with a Turkish or South Asian Surinamese background who live in disadvantaged neighborhoods span multiple interacting themes. Future studies examining the relationship between green space and health should consider interactions between motives, means, prerequisites and ethnicity. The possibility of expanding the multifunctionality of green spaces to provide marginalized populations with more equitable access and activities should be further explored.

## Introduction

Cardiometabolic diseases are the leading cause of death worldwide and a major contributor to morbidity, with their burden continuing to grow [1, 2]. In 2019, an estimated 34.4 million disability-adjusted life years were attributed cardiovascular diseases [1]. Additionally, in 2021, an estimated 79.2 million disability-adjusted life years were attributed to diabetes, mostly type 2 [2]. Throughout Europe, including in the Netherlands, populations with a Turkish or Surinamese background bear a higher cardiometabolic risk compared to the majority populations [3–6]. These populations are underrepresented in research, yet investigating factors contributing to their health inequalities is essential for building healthy and just societies [7]. In the Netherlands, the Turkish and Surinamese populations are the first and third largest immigrant populations, respectively, and they reside in cities more often than the majority Dutch population [8].

There is growing evidence that within cities the presence of green spaces such as parks contributes to better cardiometabolic health [9–12]. While its pathways remain to be fully elucidated and might differ between contexts, there is evidence of green space encouraging physical activity, social interactions, reducing stress and reducing exposure to air pollution [10, 12, 13]. Still, the health benefits of green space seem to be more significant for men than for women and differ between subpopulations at the intersections of gender, socioeconomic position, and ethnic background [9, 14].

Indeed, access to and use of green space differs by social characteristics at the level of neighborhoods, communities, and individuals. For instance, quantitative studies have shown that more deprived neighborhoods have less green space [15, 16]. Survey-based studies have also shown that minority ethnic groups experience more barriers to visit green space [17–20] and the barriers and motives for visiting green space differ between ethnic groups [17, 19, 20]. In addition, a mixed-methods study found that women visit green space less often [21]. However, most research on the use of green spaces by social characteristics has been conducted in the United States. Results cannot be extrapolated to the European context mainly because of differences in sociodemographic and environmental factors. Additionally, very few studies have been able to provide insight into the context-specific lived experiences of minority ethnic women and their motives for using green spaces, the ways in which they used green spaces (i.e.,, means) and what they need in order to use green spaces (i.e., prerequisites). A qualitative study design is most appropriate to capture these in-depth insights, which can be used to better inform locally informed policies and interventions to stimulate green space usage and prevent CVD.

Most studies on green space and behavior focus on majority populations, leading to an underrepresentation of marginalized groups, including women with Turkish or Surinamese backgrounds living in Europe. Consequently, evidence on their motives for using green spaces is not yet available. Addressing this knowledge gap can provide insight into how green space influences cardiometabolic health inequalities. In this study, we aimed to explore the motives, means and prerequisites to visit green space of women with a Turkish or South Asian Surinamese background residing in disadvantaged neighborhoods.

## Methods

### Study design

We conducted a case study through six focus group discussion (FGD) sessions as part of a larger qualitative research project on cardiometabolic disease prevention among ethnic groups [22]. This approach allowed new motives, means and prerequisites to emerge through group discussions, with the moderator playing a relatively small role. Since several of the participants had participated in group activities before, FGDs likely provided the most comfortable and encouraging setting.

The study was co-developed with three local community experts. SN, a Dutch Surinamese Hindustani man, is professionally involved in the South Asian Surinamese community in The Hague through the Vobis Foundation, a non-profit foundation that supports marginalized groups towards social and economic independence and promotes social cohesion. LJ, a Dutch Surinamese Hindustani woman, is professionally involved in the South Asian Surinamese community in The Hague through the Ester Foundation. This foundation is involved in discussion groups and support for social problems. SN and LJ have ample experience in organizing and leading information and intervention groups. CD, a Dutch Turkish woman, is professionally involved in the Turkish community in Utrecht through the Al Amal Foundation and has experience leading focus group discussions. The Al Amal Foundation focuses on improving the well-being of residents with a migration background.

Community experts (SN, LJ, CD) and three Dutch female researchers (LvdB, HV, LW) co-designed the participant recruitment, set-up and conduct of FGDs and data interpretation. Community experts were the main moderators for the focus groups, and while the coding and thematic analysis was the main responsibility of the researchers, the overall interpretation of results was conducted collaboratively between the researchers and the community experts. The community experts received monetary renumeration for their work.

### Research team and reflexivity

Since the researchers do not share the Turkish or South Asian Surinamese ethnicity and therefore do not have the same lived experiences, collaboration with local community experts was essential in ensuring that the research remained equitable and relevant to the study population [23]. By involving the community experts, we aimed to enhance cultural sensitivity throughout the research process and to foster appropriate behavior towards participants, which can reduce the burden of participating, increase trust and open communication and enrich the data [23]. The involvement of community experts in interpreting the results was particularly crucial for accurately understanding and reflecting on the participants’ perspectives and experiences within their cultural contexts, thereby minimizing potential bias stemming from the researchers’ positionality and backgrounds.

### Study setting

The study was carried out in The Hague (∼550,000 inhabitants) and Utrecht (∼360,000 inhabitants), which are the third and fourth largest cities in the Netherlands. Moerwijk (The Hague) and Kanaleneiland (Utrecht) were the starting points of the study as the community experts are involved in these neighborhoods. The FGDs took place in community centers in each neighborhood where the foundations regularly organize meetings. Kanaleneiland borders park Transwijk (area ± 45 ha). Moerwijk borders the Zuiderpark (area ± 100 ha), and vegetated dunes and the beach of the North Sea coast are at ± 5 km distance.

Life expectancy in these neighborhoods is lower than the average for the cities in which they are located [24, 25]. The percentage of households with an income below the social minimum, which was 1701 euros gross per month in 2021, was 15% in Kanaleneiland compared with 8% in all Utrecht and 23% in Moerwijk compared with 12% in all The Hague in 2021 [26]. A relatively high proportion of the residents in these two neighborhoods have a migration background: 72% in Kanaleneiland and 77% in Moerwijk [26].

### Source population and participant recruitment

Women aged 18 years or older with a self-identified Turkish background living in Utrecht or with a self-identified South Asian Surinamese background living in The Hague, were eligible for participation. The community experts recruited eligible women through the foundations by contacting them via phones calls or text messages. Information on the study was provided to potential participants on a one-page brochure. Although not a strict criterion, recruitment of participants with a self-reported history of cardiovascular disease (CVD), type II diabetes mellitus, obesity, metabolic syndrome, rheumatoid arthritis, hypertension or hypercholesterolemia was prioritized. The participants did not depend on the community experts for social aid or consent to partake in the Foundations’ activities. All participants received a gift card worth 15 Euros.

### Data collection

Data was collected in May and June 2022 through six FGDs. Although the goal was to conduct two FDGs per ethnic group, the recruitment strategy was so effective that we conducted four FGDs with Surinamese women. The groups ranged in size from 8 to 11 participants, each participating once. Audio recordings of the FGDs were made with the permission of the participants. Participants mentioned their age, neighborhood, and whether they had an elevated CVD risk yes/no. The latter we defined as one or more of the following: a self-reported history of CVD, type II diabetes mellitus, obesity, metabolic syndrome, rheumatoid arthritis, hypertension or hypercholesterolemia. Whether the participants had children, lived in single-person households, and were foreign-born was later summarized at group level by the community experts (SN, CD).

In preparation of the FGDs, multiple meetings between the researchers (LvdB, HV, LW) and community experts (SN, LJ, CD) took place to jointly refine the discussion guide. The South Asian Surinamese FGDs were led by LJ supported by SN. One of the three researchers posed in-depth questions and provided explanations where necessary. These FGDs were held in Dutch, but community experts occasionally translated into Sarnami-Hindustani for clarification. The Turkish FGDs were conducted in Turkish, led by CD and supported by one of the three researchers. CD provided the researcher a Dutch summary of the discussion at the end of each topic so that follow-up questions could be posed by the researcher.

Each FGD lasted 90–120 min. The first 60–90 min covered lifestyle behaviors and the influence of social connections [22]. Thereafter, the motives and barriers to visit green space were discussed. Green space is most commonly defined as vegetated areas, although the definitions used in the literature vary widely [27]. The following definition of green space was given to the participants: “Green space can help with healthy living. You can think of green space as the dunes, the Zuiderpark (both The Hague), park Transwijk (Utrecht) or other green spaces in the neighborhood.” Opening questions were: “Do you ever visit green space?” “What are you usually doing there?” “What is attractive about it?” “What holds you back?” “How can green space be improved to make it more attractive?” The following probes were provided: motivation, socializing, facilities, access, safety, attractiveness. These were based on previous papers summarizing the characteristics of urban parks associated with its use and physical activity [28, 29].

### Data analysis

Median (IQR) age of the study population was calculated. Frequencies (%) were reported for elevated CVD risk (yes/no) and neighborhood of residence. The number (%) of missing values was reported. Additional demographics summarized by the community experts were reported as percentages on group level. The audiotapes were transcribed verbatim and anonymized. The Turkish transcripts were translated to Dutch using the free version of DeepLTranslator.com. The researchers checked the translations and consulted CD for clarification of ambiguities. A thematic analysis was used to analyze the qualitative data [30]. Transcripts were inductively coded using the qualitative content analysis software NVivo version 12.7.0. First, one transcript was independently open-coded by three researchers (LvdB, HV, LW). This was done by selecting relevant excerpts from the transcription and attaching a keyword to this excerpt. The keywords were discussed among the three researchers to form an initial shared understanding of motives, means and prerequisites for visiting green spaces and to draft an initial code book. The five remaining transcripts were coded by two researchers based on the initial codebook but supplemented with newly emerging or re-phrased codes. Inconsistencies were solved through discussion and re-coding. Categorization and thematization of codes were based on discussions between LvdB, HV and LW. Following a number of different organizations of the codes, they were categorized into the final subthemes and themes. Any green space attributes (i.e., environmental factors) mentioned by participants, e.g., benches as means of using a park, were thematized according to the categorization of park attributes by McCormack et al. [28].

## Results

### Participant and neighborhood characteristics

In total, 46 women participated in six FGDs, including 16 with a Turkish background (2 FDGs) and 30 with a South Asian Surinamese background (4 FDGs) (Table 1). Almost all women were born in either Türkiye or Suriname (± 90%). The remaining participants were born in the Netherlands but one or both of their parents had migrated from these countries. The women with a Turkish background had a median (IQR) age of 51 (45–60) years while the women with a South Asian Surinamese background had a median age of 65 (58–67) years. More than half of the participants had a single-person household and almost all women had one or more children (± 90). Of all women, 57% reported a CVD event in the history or one or more risk factors for CVD.

**Table 1:** Participant characteristics

	South Asian Surinamese	Turkish
	*N* = 30	*N* = 16
Age in years *–* median (IQR)	65 (58–67)	51 (45–60)
*Missing – N (%)*	9 (30)	0 (0)
Elevated CVD risk (Y/N) – N (%)^1^	18 (60)	8 (50)
*Missing – N (%)*	2 (7)	2 (13)
Born in Suriname or Türkiye – (estimated %)^2^	(± 90)	(± 80)
Single-person household – (estimated %)^2^	(± 80)	(± 50)
Having one or more children – (estimated %)^2^	(± 90)	(± 90)
Neighborhood of residence:		
Moerwijk (The Hague)	9 (30)	0 (0)
Kanaleneiland (Utrecht)	0 (0)	12 (75)
Disadvantaged neighborhood - N (%)^3^	23 (77)	16 (100)
*Missing – N (%)*	2 (7)	0 (0)

IQR = interquartile range

1 defined as self-reported history of a cardiovascular event, type II diabetes mellitus, obesity, metabolic syndrome, rheumatoid arthritis, hypertension or hypercholesterolemia

2 summarized on group level by the community experts (SN, CD)

3 determined by a higher % of households with income below the legal minimum wage compared to the average of the respective city

In the Turkish FGDs, almost all participants lived in the neighborhood Kanaleneiland (75%). Of the South Asian Surinamese participants, the largest proportion (30%) lived in the neighborhood Moerwijk. 85% of all participants lived in a disadvantaged neighborhood, meaning a neighborhood with a higher-than average proportion of inhabitants with an income below the social minimum.(26) The green spaces discussed were mostly Park Transwijk in Utrecht, and Zuiderpark in The Hague. Participants visited forests, community gardens, dunes, and cemeteries as well.

### Motives, means, and prerequisites to visit green space

Four main overarching themes emerged from the results: social interactions, personal factors, activities, and environmental factors. Within these themes, 13 subthemes were identified, wherein the factors described by the participants often functioned as a motive in itself to visit green space. In addition, means to achieve outcomes within other (sub)themes often emerged, for example exercising facilitated socializing and improved physical and mental well-being. Furthermore, some prerequisites were identified, meaning that if these factors were not adequately provided, individuals would or could not visit green spaces.

### Social interactions

Socializing was an important motive to visit green space. Green space facilitated social encounters and the activities performed in green space were often carried out together. Visiting green space together functioned as a motive to walk but also served as opportunity to socialize.

*“The park is good for your social life because you occasionally run into people.” (Surinamese FGD number 2*, *[abbreviated: S2])*

Some preferred walking alone, at their own pace, but this also restricted route choices due to feeling unsafe because of darkness or poor lighting.

*“I usually walk on the main road*, *because if something happens then someone is on the street. If I am with a group*, *then I go [to the park].” (S4)*.

### Personal factors

Having time, mental well-being and physical well-being were personal factors playing a role in the motivation to visit green space. Having not enough time resulted in visiting green space less often, only visiting green space that was nearby, or not visiting green space at all.

*“I sometimes take walks but not really through the forest. I don’t have that much time for that.” (S1)*.

*“Not very often because I have a pretty busy schedule.” (S2)*.

Mental well-being was more often mentioned than physical well-being as a motive to visit green space. Stress reduction, happy feelings, and enjoying the surroundings were motivating and rewarding sensations.

*“I enjoy nature when I walk in a park or on the dunes*, *I enjoy the sea and the sky and the trees because it gives you me much peace. When I walk there*, *I don’t look at people*, *I look at those trees and those birds and everything.” (S3)*.

*“It gives peace of mind*, *makes you feel relaxed.” (Turkish FGD number 1 [abbreviated T1])*

Physical well-being was both a motive and a prerequisite to visit green space. The physical health promoting effects of visiting green space were perceived to operate through exposure to fresh air and through stimulating physical activity.

*“The more you exercise*, *the more muscular you are*, *the less you age.” (S2)*.

*“The forest provides fresh air. It is very healthy.” (T2)*.

A synergistic effect on physical and mental well-being in relation to visiting green space frequently emerged (B1).

*“It is good for your mind and for your body.” (S1)*.

Having to pee often in relation to health conditions or having hay fever symptoms were barriers to go outside and visit green space.

### Activities in green space

Undertaking activities was sometimes a motive in itself for visiting green space, but often acted as a means to pursue the benefits of green space visits within the other themes, such as social interactions (walking or picnicking together), physical well-being (exercising) and mental well-being (exercising, relaxing).

*“Being active with other people. You see each other. You see people you [normally] don’t see.” (T1)*.

The type of activity carried out depended on environmental factors, including the availability of sports equipment, benches and toilets.

*“We go to park Transwijk*, *but we can’t stay there all day if there is no toilet. We would like to spend the whole day there on Saturday and Sunday.” (T1)*.

*“That’s why we can’t go on picnics. When we go*, *some of our ladies are afraid to drink water because they think they will go to the toilet. That is why they do not visit.” (T1)*.

While walking through green space was very important among the South Asian Surinamese women, Turkish women mentioned more variety of activities such as picnicking and longer recreation in green space in addition to physical activity.

*“There should be picnic areas. There should be a sink next to the picnics*, *then a hand-washing area*, *a toilet and barbeque area.” (T2)*.

### Environmental factors

Characteristics of the environment (facilities, maintenance, distance, aesthetics, and safety) were reported as more relevant by the Turkish women than by the South Asian Surinamese women. In particular, facilities, aesthetics, and safety were discussed and compared to the better-equipped and better-maintained green spaces in Türkiye.

*“There should be toilets and fountains everywhere like in Turkey.” (T1)*.

*“Sports equipment like in Turkey.” (T2)*.

### Facilities

Picnic areas, barbecues, playgrounds, and sports equipment emerged acted as means to perform various activities and socialize. The presence of toilets, running water and benches was often mentioned as an unmet prerequisite to visit green space.

*“If I really need to*, *I go to the toilet in the library or at the KFC*, *but I walk*, *but not through the park because there’s nowhere to go there.” (S2)*.

*“There must be a toilet and a place to wash your hands*, *it must be clean.” (T2)*.

The lack of well-maintained toilets was a major barrier for women who needed to go to the toilet more often due to physical health problems and for (Turkish) women that wanted to visit green space for a longer time.

*“We go to the park*, *but we cannot stay there all day if there is no toilet. Because people with health problems often need to go to the toilet.” (T1)*.

### Maintenance

Comments on maintenance were mostly related to the cleanness of toilets, lack of running water, dog waste and litter lying around. This was perceived as bothersome.

### Distance

All participants thought green space was available nearby. However, desired facilities were sometimes lacking in these green spaces. The time investment and need for a car to reach more distant green spaces that do contain such facilities were barriers to go there.

*“I am looking for a park with a fountain and picnic areas. Sometimes that is far away. Everyone needs a car to get to such a place.” (T2)*.

### Aesthetics

Flowers, trees, grass fields, and especially a mix of these were considered appealing. Enjoying the aesthetical appeal of green space was a means of improving mental well-being.

*“It’s very relaxing. I get completely absorbed by the beautiful surroundings with the beautiful trees. Sometimes there are those beautiful bushes. I feel wonderful.” (S2)*.

Trees, in part because they provide shade, were desired and some women preferred to visit forests over parks. Some women with a South Asian Surinamese background regarded the attractiveness of green space in the Netherlands poor compared to less manicured nature in Suriname.

*“There is real forest in Suriname. But here they have planned it so in Suriname I feel more comfortable [in nature].” (S4)*.

### Safety

Safety was often mentioned as prerequisite for visiting green space. Safety barriers were mostly social in nature and related to alcohol and drug use, vandalism, and darkness. Unsafe feelings depended on the landscape, as shrubs blocked sightlines and therefore created uncertainty and unsafe feelings. Reducing the density of shrubs was suggested to improve safety.

*“Less tall plants and less dense planting so I can see through them.” (S4)*.

Visiting green spaces together increased sense of safety as well. Other proposed measures included increasing the number of streetlights, security cameras, surveillance, and police.

## Discussion

Our qualitative study with women with a Turkish or South Asian Surinamese background living in disadvantaged neighborhoods in the Netherlands showed that the wish to socialize and improve mental and physical well-being were, often in combination, important motives to use nearby green spaces. Undertaking activities (walking, picnicking) and interacting with facilities (sports equipment, barbeque areas) were means to socialize and pursue well-being. Several unmet prerequisites for visiting green space were mentioned, such as well-maintained toilets, safety, having time, and physical well-being. There were notable interactions between motives, means and prerequisites. For instance, visiting a park together with others influenced the activities that were undertaken and provided a feeling of safety. Although motives, means, and prerequisites to visit green space were largely similar between ethnic groups, Turkish women mentioned a wider range of activities, and facilities played a more prominent role compared to South Asian Surinamese women.

### Overall comparison of motives, means and prerequisites to other studies

Most previous qualitative studies on green space usage focused on certain environmental characteristics, and whether those acted as facilitators or barriers [17, 28, 31]. Our results within the theme environmental factors corroborate their findings. In addition, our participants mentioned that socializing, mental well-being, physical well-being, and exposure to fresh air were important motives to visit green space. However, the literature evaluating these factors in relation to green space use is heterogeneous in terms of geographic regions, environmental characteristics, methods, populations studied, and methods used to define ethnic groups. This hinders a general comparison with the motives we have identified.

#### Population characteristics related to motives, means and prerequisites

The slight differences between the ethnic groups in our study were particularly evident in the comparisons made with green space in Türkiye and Suriname: while Turkish participants expressed a desire for better facilities in green space as present in Türkiye, Surinamese participants longed for preserved nature as present in Suriname. A study in Türkiye also reported a broad range of recreational activities performed in urban green spaces, which is in line with preferences of our participants with a Turkish background [32]. Please note that the median age of the Turkish participants was lower than of the South Asian Surinamese participants in our study, which could have played a role in the identified differences.

Previous studies on ethnic differences in motives to visit green space were predominantly survey-based, quantitively analyzed and performed in other geographical contexts and populations than our study, limiting comparability [17–19]. Nonetheless, congruency in motives for green space visitation across ethnic groups was found in these studies as well. Although differences were also reported, including group size during visits, the amount of barriers and benefits experienced and preferences for physically active visits [17–19].

Socializing was a very important motive for our female participants. That is in line with previous studies showing that socializing in green space was a more important motive for women than for men [28], with the latter using green space more often and performing more physical activity than women [33], especially in disadvantaged neighborhoods [34].

In our study, safety concerns were a barrier to visit green space especially when alone or in the dark. This may be related to the characteristics of our study population, as other studies have indicated that unsafety was a bigger barrier to visit green space for women compared to men [28, 35], and for non-Hispanic Blacks and Hispanics compared to non-Hispanic whites [17]. It is unknown to what extent the latter was caused by more unsafe green space. A systematic review into fear of crime in green spaces found more fear among women, among minority ethnic populations and especially among women from minority ethnic populations and low-income minority ethnic populations [36]. Our participants mentioned multiple modifiable factors to increase safety including increasing streetlights and increasing sight lines by using more open natural features.

### Links between motives, means and prerequisites

We found interactions between various motives, means and prerequisites within and across themes, especially for the theme social interactions. Two previous studies also showed that socializing is a motive to perform physical activity, especially for people who do not regularly exercise [37, 38]. For some social activities in our study such as picnicking, a picnic area with many benches and running water was a prerequisite that was currently not met. A study on green space visitations in Norway concluded something similar, namely that including designated places for social interactions, such as benches, can increase the use of green space [39]. We noticed that many of the motives, means, and prerequisites that emerged from our study overlap with mediators identified in quantitative studies on the relationship between green space and health, particularly physical activity, socializing, and stress reduction [12, 40, 41]. The links we identified between these themes suggest interrelationships in the pathways from green space exposure to health and the difficulty to distinguish these causal pathways.

Other crosslinks were seen between green space visitation, aesthetics, and mental and physical health. In line with previous findings that the quality of green space determines its benefits, we found that the positive effects of green space visitation on mental well-being depend on the aesthetic attractiveness of the green space [42]. Other studies showed that natural features such as trees, water and shrubs and less paved spaces in urban green spaces have restorative benefits, relieve stress and promote positive emotions and feelings [43]. Our participants also mentioned a combined motivation of mental and physical well-being to visit green space. Other studies confirm the relation between green space, physical activity, and mental and physical health [40, 44, 45]. The relation between less dense planting to increase sight lines and perceived safety was mentioned by our participants like in previous studies [28].

#### Strengths and limitations

This study is the first to investigate the motives and barriers to visit green space among Turkish and South Asian Surinamese women in the Netherlands, who are underrepresented in research and have a high CVD risk. The qualitative design of the study allowed us to investigate the interplay between the identified motives, means and prerequisites. Carrying out this study in co-creation with local community experts had several advantages. It enabled us to reach these underrepresented communities. As collaboration with the community experts happened throughout all stages of research, biases resulting from positionality and privilege of the researchers were minimized. Because the participants often knew the community experts beforehand, the discussion sessions were held in a comfortable and familiar environment. On the one hand, this familiarity may have encouraged more open sharing of information. On the other hand, some participants may have been hesitant to disclose sensitive information to prevent that the community experts would change their views on them. The FGDs with Turkish women were conducted in Turkish, which limited the direct involvement of co-moderators who did not speak the language. We believe this did not affect the study’s credibility, as CD, who is highly fluent in both languages, provided nuanced summaries of participants’ responses, allowing the co-moderators to ask in-depth questions. Moreover, conducting the sessions in the participants’ native language enhanced inclusiveness and facilitated more effective communication. The use of multilingual transcription professionals further ensured accurate transcription and translation. We were unable to compare the results with barriers and motives experienced by other ethnic groups and men, which limits our options to conclude whether the identified motives, means and prerequisites are specific to the groups studied. Furthermore, the FGDs were not solely dedicated to the topic of green space usage. Participants may already have been nudged towards certain topics after the first two discussion topics, and there was no time for elaborate in-depth follow-up discussions.

#### Implications

The wide range of motives, means, and prerequisites and many interactions observed, underpins the need to increase the multifunctionality of green spaces and improve its community-inclusiveness [46, 47]. While the specific motives, means and prerequisites are likely to differ between geographical and social contexts, involving (ethnic) minority groups in plans for the design, maintenance and renovation of green spaces are likely to result in their broader use. In addition, improving green space maintenance as a facilitator for increased usage seems like low-hanging fruit. Future studies should explore whether interventions targeted at the identified motives, means, and prerequisites increases green space access for marginalized populations and decreases health inequalities. Such studies must involve various stakeholders including community experts and city planners. Future studies into the association between green space exposure and health (behaviors) should take into account interactions between motives, means, and prerequisites, and effect modification by ethnicity.

## Conclusion

The motives, means and prerequisites to visit green space of women with a Turkish or South Asian Surinamese background living in disadvantaged neighborhoods in the Netherlands span multiple interacting themes. We found an interplay between physical well-being, mental well-being, socializing, and environmental factors in their effect on the motivation to visit green space. Future intervention studies should target these and evaluate whether they benefit marginalized populations. Future studies examining the relationship between green space and health behaviors should take into account interactions between motives, means, prerequisites, and effect modification by ethnicity.

## Data Availability

No datasets were generated or analysed during the current study.
